# Subungual Verruca Vulgaris Mimicking Onychomycosis

**DOI:** 10.7759/cureus.55085

**Published:** 2024-02-27

**Authors:** Kawaiola Cael Aoki, Summer Wong, Simona Bartos

**Affiliations:** 1 Dr. Kiran C. Patel College of Osteopathic Medicine, Nova Southeastern University, Fort Lauderdale, USA; 2 Department of Dermatology, Imperial Dermatology, Hollywood, USA

**Keywords:** subungual lesion, subungual location, wart treatment, verruca vulgaris, nail dystrophy, nail plate, onychomycosis, nail diseases

## Abstract

Nail abnormalities, or onychodystrophy, can be caused by various pathologies, including fungal and nonfungal infections. These can result in difficulties with nail trimming, pain, and social discomfort that can significantly impact a patient's quality of life. Even experienced physicians may find it challenging to diagnose due to the lack of specificity in these changes. We present the case of a 60-year-old female who was initially diagnosed with onychodystrophy but was later found to have subungual verruca vulgaris after a nail avulsion and biopsy. This case highlights the importance of thorough diagnostic procedures and considering a broad range of differential diagnoses. We also discuss the challenges of treating subungual warts and the need for a precise therapeutic approach to ensure the best possible outcomes.

## Introduction

Atypical nail presentations can be attributed to fungal and nonfungal infections, inflammatory dermatological disease, malnutrition, neoplastic growth, and trauma. Onychodystrophy denotes deviations in nail morphology beyond discoloration or nail dyschromia. Nail fragility, or brittleness, is attributable to the partial or complete disruption of the various keratinous strata constituting the nail plate [1]. Routine nail examinations during patient consultations are essential, as they allow clinicians to detect systemic manifestations through subtle changes in the nail unit’s anatomy, including the nail matrix, nail plate, nail bed, and vasculature [2]. The nonspecific nature of such changes presents a diagnostic challenge for even the most experienced physicians.

Onychodystrophy can drastically impact an individual's quality of life, and fungal infections account for about 50% of cases [1,3]. Onychomycosis, in particular, exhibits overlapping signs and symptoms with other conditions, including psoriasis, trauma, and lichen planus [3]. These patients may face challenges such as nail trimming difficulties, discomfort/pain, sensations of nail pressure, and feelings of social embarrassment. Additionally, patients experiencing treatment failure, improvement, or even cure continue to experience impairment in physical functionality, emotional health, and social interactions [1].

On the other hand, wart infections are caused by the human papillomavirus (HPV) and are most commonly found on the hands, feet, and elbows [4]. Less frequently, HPV can infect subungually or beneath the nail plate. These warts can initially manifest as small lesions and multiply, leading to more extensive, hyperkeratotic growths called verruca vulgaris [4]. As the size of the wart increases, it can progressively deform the shape of the nail plate, causing pain and discomfort [5]. Here, we document a case that illustrates how an initial diagnosis of onychodystrophy was later revised to a subungual verruca vulgaris after a subsequent nail avulsion and biopsy.

## Case presentation

**Figure 1:**
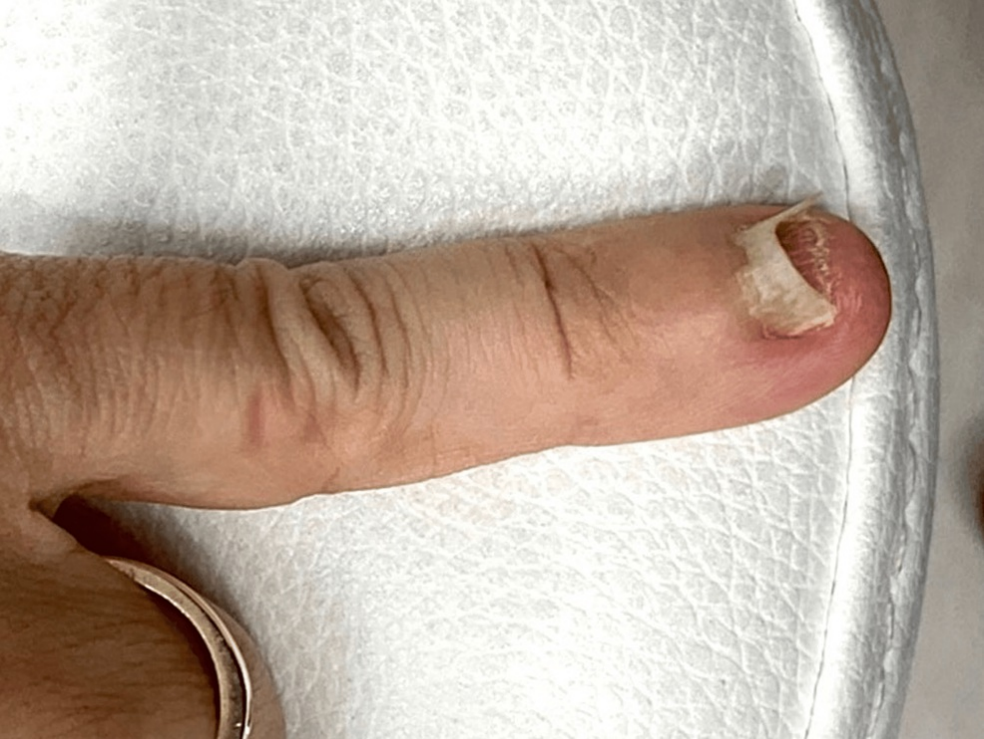
Atypical nail of the fifth digit on initial presentation

A 60-year-old female presents with severe nail damage and yellowing of the left fifth digit for several months. She has no systemic complaints or comorbidities. Changes to the nail plate concerning underlying malignancy or infection were seen on the physical exam (Figure 1).

**Figure 2:**
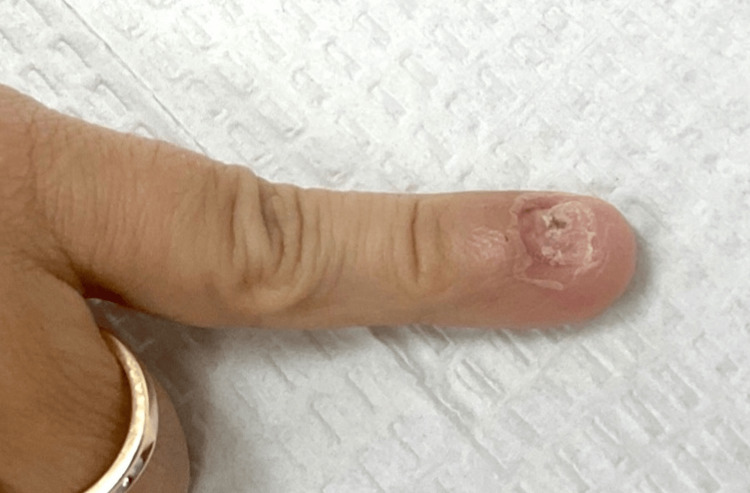
Subungual verruca vulgaris after nail avulsion

A biopsy via nail clipping was performed to test for fungal infection. Biopsy results showed hyperkeratosis consistent with onychodystrophy but stained PAS negative for pathogenic fungi. Anterior-posterior and lateral radiographs were obtained to rule out soft tissue tumors such as onychomatricomas, glomus tumors, or subungual exostosis. The nail was dorsally offset, but no exostosis or significant bone or joint space pathology was appreciated. The nail continued to show thickness, brittleness, crumbling, and an irregular lesion at follow-up. A nail avulsion was performed, and a nail clipping and shave biopsy of the lesion were sent to pathology. Differential diagnoses included onychomatricoma, onychomycosis, and onycholysis due to trauma. The pathology indicated an irritated verruca with incidental fungal elements secondary to nail trauma. The healing digit after the nail avulsion can be seen in Figure 2.

**Figure 3:**
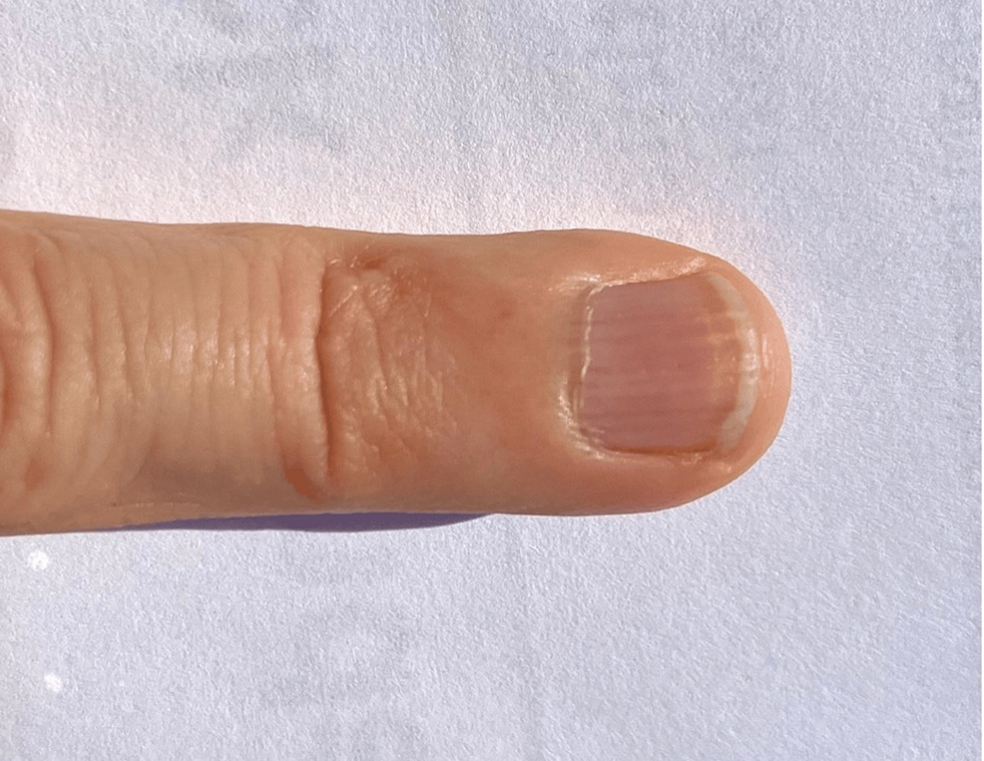
Complete nail regrowth seven months after nail avulsion

The verruca vulgaris was destroyed by paring with a 15-blade, followed by liquid nitrogen cryotherapy (three, three-second freeze-thaw cycles). She was prescribed wart remover (40%) adhesive patches to continue treatment for one month. At seven months after the avulsion, the patient reported complete nail regrowth and no recurrence of the wart (Figure 3).­

## Discussion

The gold standard for diagnosing onychomycosis is culture, but no standard exists for HPV lesions. Diagnostic options include histopathological examination, ultrasound, dermoscopy, and polymerase chain reaction [3]. The differential diagnosis for common warts, particularly when they manifest subungually, can raise concerns about malignant growth, such as squamous cell carcinoma or Bowen’s disease. Thus, it may be advisable to promptly perform a surgical excision and biopsy of any suspicious growths underneath the fingernail. Other potential differential diagnoses include basal cell carcinoma, malignant melanoma, subungual exostosis, pyogenic granuloma, fibromas, epidermoid carcinomas, and keratocanthomas [4].

Subungual warts represent a particularly aggressive variant of common warts since they are resistant to conventional treatment approaches, and these may often result in further injury to the nail matrix and permanent nail deformity [5]. Treatment options for warts include topical salicylic acid, laser cauterization, cryotherapy, thermotherapy, photodynamic therapy (PDT), and surgical excision [6]. However, the nail’s barrier properties pose a challenge with treatment, as many medications cannot penetrate the keratinized tissue effectively [5]. Due to these properties, surgical removal is recommended, especially considering that certain HPV infections increase the risk of squamous cell carcinomas [4,5]. Several studies have also demonstrated promising outcomes with using PDT in treating subungual warts for its ability to selectively destroy epidermal warts while simultaneously preserving the dermis, mitigating adverse effects such as ulceration and scarring [6,7]. However, a definitive cure is not guaranteed following any therapy; the risk of recurrence is high, and multiple treatment sessions are often needed. Differentiating between various nail presentations requires a high degree of clinical suspicion and is essential to directing therapeutic strategies to improve patient outcomes.

## Conclusions

This case underscores the importance of considering a wide range of differential diagnoses for nail abnormalities, as these can be attributed to various underlying pathologies. Subungual verruca vulgaris, although rare, can mimic onychomycosis and should be considered when other treatments fail. The challenging nature of diagnosing nail disorders and their impact on patient quality of life requires improved diagnostic accuracy and a precise treatment approach for optimal patient outcomes and to prevent drug resistance. Clinicians should remain vigilant in differentiating between nail disorders, especially in cases of subungual growth, to provide the most appropriate and effective care.
